# Guardian’s knowledge and attitude towards inhaled corticosteroids aerosol therapy and medication compliance of children with wheezing diseases

**DOI:** 10.1186/s13223-024-00908-5

**Published:** 2024-07-24

**Authors:** Zuojiao Liu, Haiqing Dai, Fengjiao Tao, Xiaoxiao He, Ting Jin

**Affiliations:** The First Affiliated Hospital of Shaoyang University, Shaoyang, 422003 China

**Keywords:** Wheezing disease, Inhaled corticosteroids, Asthma, Medication compliance, Knowledge attitude practice

## Abstract

**Background:**

Glucocorticoids are widely used in inhalation aerosol therapy for wheezing diseases. This study aims to explore guardians’ knowledge and attitude towards inhaled corticosteroids (ICS) aerosol therapy and the medication compliance of children with wheezing diseases in China.

**Methods:**

This cross-sectional study enrolled guardians of children with wheezing diseases at the First Hospital Affiliated to Shaoyang College between October 2022 and February 2023. A self-administered questionnaire was developed to collect demographic information of the participants and evaluate their knowledge and attitude towards ICS aerosol therapy. The 8-item Morisky Medication Adherence Scale was used to assess the medication compliance of children.

**Results:**

A total of 506 valid questionnaires were collected. 260 (51.38%) participants were guardians of a ≤ 3-year-old child and 327 (64.62%) were children’s mothers. The knowledge, attitude, and medication compliance scores of all participants were 12.61 ± 5.78, 20.95 ± 2.37, and 4.69 ± 2.18, respectively. Multivariate logistic regression showed that knowledge scores [OR = 1.053, 95% CI (confidence interval): 1.017–1.090, *P* = 0.003], attitude scores (OR = 1.121, 95% CI: 1.030–1.219, *P* = 0.008), guardians of children aged 4–6 years (OR = 0.385, 95% CI: 0.242–0.612, *P* < 0.001), and grandparents of children (OR = 2.633, 95% CI: 1.104–6.275, *P* = 0.029) were independently associated with children’s medication compliance.

**Conclusions:**

In conclusion, guardians of children with wheezing diseases in China had insufficient knowledge, unsatisfactory attitude, and poor medication compliance towards ICS aerosol therapy.

**Trial registration:**

Retrospectively registered.

## Background

Wheezing disease is one of the most common respiratory diseases in childhood, and was classified into transient wheeze, early persistent wheeze, and late onset asthma [1, 2]. Approximately 34% of children have had wheezing experiences before the age of three, and 50% of children would have it before the age of six [3]. The clinical manifestations of the child are wheezing, coughing, and shortness of breath. Many factors can cause children’s wheezing, including infections, allergies, and a dusty environment [4, 5]. Recurrent wheezing diseases in childhood can greatly influence the immune and respiratory systems of children, increasing the risk of late onset asthma and respiratory failure [6], thus bringing heavy burdens to families and society [7, 8].

Due to the complex pathogenesis of wheezing diseases, variation exists regarding the choice of therapy for children [9, 10]. Currently, inhaled corticosteroids (ICS) aerosol therapy has been recognized as the preferred anti-inflammatory drug for the long-term treatment of wheezing diseases and the main drug for treating moderate to severe asthma in adults and children worldwide, and its safety and effectiveness have been widely proven [11]. Daily ICS therapy can prevent exacerbations in recurrent wheezing diseases. The intermittent ICS is also useful for preventing exacerbations of intermittent asthma or viral-triggered wheezing [12].

Studies on continuous use of ICS showed patients had low persistence and adherence rates varying from 17 to 60% [13]. A large-sampled cohort study reported the low use and poor adherence to ICS medication in children/adolescent asthma patients [14]. Poor adherence is associated with a lack of patients’ knowledge about the disease and treatment. For pediatric patients, their parents and other guardians play a pivotal role in the management of wheezing diseases as the guardians decide whether they will follow medical advice or not. Parental decisions and medication practices are largely based on their knowledge of the illness and attitude towards the medication. Educating their guardians on self-management and the correct use of medication is a fundamental component of disease management guidelines [13].

Previous studies in Western countries have reported poor parental knowledge of asthma and low medication compliance of patients to ICS therapy [15–17]. However, the guardians’ knowledge and attitude and children’s medication compliance to ICS aerosol therapy in China are still unclear. Besides, few studies have explored determinants of ICS medication compliance in children with wheezing diseases. Therefore, this study aimed to explore guardians’ knowledge and attitude towards ICS aerosol therapy and the medication compliance of children with wheezing diseases in China and to investigate the influencing factors of ICS medication compliance.

## Methods

### Study design and participants

This cross-sectional study enrolled guardians of children with wheezing diseases at the First Hospital Affiliated to Shaoyang College between October 2022 and February 2023. The inclusion criteria were: (1) guardians of children aged < 18 years old with a confirmed diagnosis of wheezing diseases; (2) guardians of children who underwent ICS aerosol therapy; and (3) voluntarily participated in this study. Wheezing diseases included: asthma, bronchiolitis, asthmatic bronchitis, Mycoplasma pneumoniae pneumonia, and occlusive capillary bronchitis [18–20]. The exclusion criteria were: (1) guardians of children receiving long-term systemic immunosuppressive therapy; (2) guardians with cognitive impairment or inability to complete the questionnaires; and (3) guardians considered unsuitable for this study for other reasons. This study was approved by the Medical Ethics Committee of the First Affiliated Hospital of Shaoyang University (k2022-014-01), and informed consents were obtained from all participants.

### Questionnaires

The self-administrated questionnaire was designed referring to previous studies and Expert consensus on the use of aerosol therapy of inhalation glucocorticoids in pediatrics [21, 22], and was modified by a senior expert in pediatrics. A total of 101 guardians were randomly selected for reliability and validity test, and the Cronbach’s α and Kaiser-Meyer-Olkin (KMO) value of the questionnaire were 0.8840 and 0.7895, respectively.

The final questionnaire contained 4 dimensions: demographic information section (including the children’s age, gender, guardian’s education, guardian’s relationship with the child, child’s healthcare payment, child’s type of wheezing disease), knowledge dimension, attitude dimension, and medication compliance dimension. The knowledge dimension consisted of 13 items and 3 questions, of which Questions 5 and 7 were multiple-choice questions and Question 10 was a single-choice question, with 1 point for a correct answer and 0 points for a wrong answer. Other items in the knowledge dimension were scored as 0, 1, and 2 points for “not aware”, “moderately aware”, and “very aware”, respectively. The total scores of the knowledge dimension ranged from 0 to 29 points. The attitude dimension consisted of 5 questions using a 5-point Likert scale ranging from strongly agree (5 points) to strongly disagree (1 point). The total scores of the attitude dimension ranged from 5 to 25 points. The 8-item Morisky Medication Adherence Scale (MMAS-8) (scoring range 0–8) was used to assess the medication compliance of children, with a score of < 6 representing poor medication compliance, and 6–8 representing moderate-to-good compliance [23].

### Data collection and quality control

Questionnaires were administered via the convenience sampling method. An online questionnaire was distributed to participants by the Sojump website (https://www.wjx.cn/), and participants filled out the electronic questionnaires via smartphones by scanning the Quick Response Code of the questionnaire. To ensure the data quality and completeness of the questionnaires, the questionnaire could be completed only once per IP address with all questions answered. Two trained research assistants assisted participants in completing the questionnaires and checked all questionnaires for completeness, internal coherence, and reasonableness. Questionnaires with contradictory logic or incomplete answers were considered invalid and excluded.

### Statistical analysis

SPSS 26.0 (IBM Corp., Armonk, NY, USA) was used for statistical analysis. Continuous data were presented as mean ± standard deviation, and categorical data were presented as cases (percentages). Continuous variables with normal distribution were compared using the Student’ t test between 2 groups and one-way analysis of variance (ANOVA) between multiple groups; continuous variables with skewed distribution were compared using Wilcoxon-Mann-Whitney test between 2 groups and Kruskal-Wallis analysis between multiple groups. The Pearson correlation analysis was used to explore the correlation between knowledge, attitude, and medication compliance. Multivariate logistic regression was performed to analyze the factors associated with guardians’ medication compliance. The variables that were clinically significant in univariable logistic regression and some additional variables, such as education and healthcare payment were included in the multivariable logistic regression. An MMAS-8 score < 6 was considered as poor compliance and a score ≥ 6 as moderate-to-good compliance. Variables with *P* < 0.05 in the univariate analysis were included in the multivariate regression analysis. A two-sided *P* < 0.05 was considered statistically significant.

## Results

A total of 510 questionnaires were collected, and four questionnaires were excluded due to contradictory logic or incomplete answers. Finally, 506 questionnaires were included and the effective rate was 99.22%. Of the enrolled participants, 286 (56.62%) were guardians of a male child, 260 (51.38%) were guardians of a ≤ 3-year-old child, 327 (64.62%) were the mothers and 84 (16.60%) were the fathers of children. A total of 270 (53.36%) participants had an education of bachelor’s degree and above, and 406 (80.24%) of their children had medical insurance (Table 1). Among the children of enrolled participants, 399 (78.85%) had bronchiolitis, 66 (13.04%) had asthma, 159 (31.42%) had asthmatic bronchitis, 58 (11.46%) had Mycoplasma pneumoniae pneumonia, 19 (3.75%) had occlusive capillary bronchitis.

**Table 1:** Baseline characteristics of the study population

Variables	*N* (%)	Knowledge		Attitude		Medication compliance	
		Score	*P*	Score	*P*	Score	*P*
**Total**	**506**	12.61 ± 5.781		20.95 ± 2.371		4.69 ± 2.181	
**Gender of children**			0.566		0.496		0.639
Male	286 (56.62)	12.48 ± 5.496		20.89 ± 2.280		4.65 ± 2.197	
Female	220 (43.48)	12.78 ± 6.140		21.04 ± 2.486		4.75 ± 2.163	
**Age of children**			0.050		0.088		0.005
0–3 years	260 (51.38)	12.00 ± 5.622		21.01 ± 2.314		5.02 ± 2.140	
4–6 years	149 (29.45)	13.32 ± 5.709		21.15 ± 2.421		4.25 ± 2.094	
6 years and above	97 (19.17)	13.14 ± 6.180		20.49 ± 2.407		4.51 ± 2.297	
**Guardian’s relationship with the children**			0.363		0.393		0.032
Father	84 (16.60)	13.31 ± 6.288		20.92 ± 2.180		4.59 ± 2.278	
Mother	327 (64.62)	12.60 ± 5.537		21.05 ± 2.398		4.75 ± 2.075	
Grandparents	33 (6.52)	11.21 ± 5.195		21.00 ± 2.398		5.49 ± 2.289	
Other	62 (12.25)	12.47 ± 6.565		20.48 ± 2.454		4.14 ± 2.422	
**Guardian’s education**			0.000		0.025		0.851
High School and below	119 (23.52)	9.76 ± 4.703		20.55 ± 2.403		4.79 ± 2.150	
Junior College	117 (23.12)	12.72 ± 5.807		20.78 ± 2.553		4.67 ± 2.142	
Bachelor’s degree and above	270(53.36)	13.81 ± 5.784		21.21 ± 2.248		4.66 ± 2.217	
**Healthcare payment**			0.004		0.001		0.220
Medical Insurance	406 (80.24)	12.98 ± 5.803		21.12 ± 2.362		4.75 ± 2.210	
Out-of-pocket Payment	100 (19.76)	11.11 ± 5.468		20.28 ± 2.297		4.46 ± 2.050	

The knowledge, attitude, and medication compliance scores of all participants were 12.61 ± 5.78, 20.95 ± 2.37, and 4.69 ± 2.18, respectively. There were significant differences in the knowledge and attitude scores between participants with different education (all *P* < 0.05). Guardians of children with medical insurance had significantly higher knowledge scores (12.98 ± 5.80 vs. 11.11 ± 5.47, *P* = 0.004) and higher attitude scores (21.12 ± 2.36 vs. 20.28 ± 2.30, *P* = 0.001) than those with out-of-pocket payment. There were significant differences in medication compliance scores between children of different ages and children of different relationships with their guardians (all *P* < 0.05) (Table 1).

In the assessment of knowledge towards ICS aerosol therapy, items with the lowest scores included “adverse effects of glucocorticoid therapy,” “medication for wheezing diseases,” and “etiology of wheezing disease.” Besides, a mere 137 (27.08%) and 127 (25.10%) participants answered correctly for “predisposing factors of wheezing diseases” and “adverse effects of ICS aerosol therapy.” In contrast, 470 (92.89%) participants answered correctly for “suitable position for ICS aerosol therapy in children.” Items with the highest scores included “at the end of inhalation therapy, the inhalation aerosol device needs to be cleaned in time and dried for future use,” “you should help your child to rinse his/her mouth for oral care and clean his/her face after inhalation aerosol therapy,” and “you should help your child to cough and cough after inhalation aerosol therapy, e.g., turning and slapping on his/her back,” with 134 (26.48%), 107(21.15%), and 91 (17.98%) participants being well aware of the items (Table 2).

**Table 2:** Knowledge of guardians towards ICS therapy

Knowledge	Unaware	Moderately aware	Very aware
K1. Classification of wheezing diseases	192 (37.94)	284 (56.13)	30 (5.93)
K2. Etiology of wheezing diseases	204 (40.32)	274 (54.15)	28 (5.53)
K3. Clinical manifestations of wheezing diseases	159 (31.42)	307 (61.67)	40 (7.91)
K4. Medication for wheezing diseases	214 (42.29)	259 (51.19)	33 (6.52)
K6. Adverse effects of glucocorticoid therapy	235 (46.44)	237 (46.84)	34 (6.72)
K8. Classification of inhalation aerosol devices	157 (31.03)	299 (59.09)	50 (9.88)
K9. Correct operation of inhalation aerosol therapy	89 (17.59)	345 (68.18)	72 (14.23)
K11. You should clean the child’s mouth and wash his/her face before inhalation aerosol therapy and should not use oily face cream.	104 (20.55)	341 (67.39)	61 (12.06)
K12. Correct breathing mode before inhalation aerosol therapy	118 (23.32)	331 (65.42)	57 (11.26)
K13. You should observe the child’s complexion and breathing during the inhalation aerosol therapy.	74 (14.62)	359 (70.95)	73 (14.43)
K14. You should help your child to rinse his/her mouth for oral care and clean his/her face after inhalation aerosol therapy.	69 (13.64)	330 (65.22)	107 (21.15)
K15. You should help your child to cough and cough after inhalation aerosol therapy, e.g., turning and slapping his/her back.	84 (16.60)	331 (65.42)	91 (17.98)
K16. At the end of inhalation therapy, the inhalation aerosol device needs to be cleaned in time and dried for future use.	43 (8.50)	329 (65.02)	134 (26.48)
	Correct rate		
K5. Predisposing factors of wheezing diseases	137 (27.08)		
K7. Adverse effects of ICS aerosol therapy	127 (25.10)		
K10. Suitable position for ICS aerosol therapy in children	470 (92.89)		

Pearson correlation analysis revealed that the knowledge scores of participants were positively correlated with their attitude scores (*r* = 0.240, *P* < 0.001) and children’s medication compliance (*r* = 0.214, *P* < 0.001), and their attitude scores were also positively correlated with children’s medication compliance (*r* = 0.098, *P* = 0.027) (Table 3). Multivariate regression analysis showed that knowledge scores [OR = 1.053, 95% CI (confidence interval): 1.017–1.090, *P* = 0.003], attitude scores (OR = 1.121, 95% CI: 1.030–1.219, *P* = 0.008], guardians of children aged 4–6 years (OR = 0.385, 95% CI: 0.242–0.612, *P* < 0.001], and grandparents of children (OR = 2.633, 95% CI: 1.104–6.275, *P* = 0.029) were independently associated with moderate-to-good medication compliance of children (Table 4). However, the guardian’s education and healthcare payment were not significantly associated with medication compliance of children.

**Table 3:** Pearson correlation analysis between knowledge, attitude, and medication compliance

	Knowledge	Attitude	Medication compliance
**Knowledge**	1		
**Attitude**	0.24 (*P* = 0.000)	1	
**Medication compliance**	0.214 (*P* = 0.000)	0.098 (*P* = 0.027)	1

**Table 4:** Univariate and multivariate analysis for medication compliance

Variables	Univariate logistic regression		Multivariate logistic regression	
	OR (95% CI)	*P*	OR (95% CI)	*P*
**Knowledge**	1.050 (1.017–1.085)	0.03	1.051 (1.014–1.089)	0.006
**Attitude**	1.144 (1.056–1.240)	0.001	1.117 (1.026–1.217)	0.011
**Gender of children**				
Male	Ref.			
Female	1.110 (0.770–1.598)	0.576		
**Age of children**,** years**				
0–3 years	Ref.		Ref.	
4–6 years	0.445 (0.286–0.693)	0.000	0.379 (0.237–0.607)	0.000
6 years and above	0.671 (0.412–1.091)	0.108	0.665 (0.399–1.109)	0.118
**Guardian’s relationship with the children**				
Father	Ref.		Ref.	
Mother	1.114 (0.671–1.850)	0.676	1.060 (0.623–1.806)	0.829
Grandparents	2.400 (1.055–5.459)	0.037	2.736 (1.110–6.742)	0.029
Other	1.024 (0.511–2.052)	0.946	0.825 (0.443–1.931)	0.835
**Guardian’s education**				
High School and below	Ref.		Ref.	
Junior College	1.027 (0.603–1.749)	0.923	0.978 (0.547–1.747)	0.939
Bachelor’s degree and above	1.078 (0.688–1.691)	0.742	1.103 (0.660–1.844)	0.707
**Healthcare payment**				
Medical Insurance	1.346 (0.842–2.151)	0.214	0.939 (0.572–1.544)	0.805
Out-of-pocket Payment	Ref.		Ref.	
**Types of wheezing diseases in children**				
1 type	Ref.			
2 types	0.838 (0.510–1.378)	0.487		
3 types and above	1.056 (0.513–2.174)	0.882		

## Discussion

This study revealed that the guardians of children with wheezing diseases in China had insufficient knowledge, unsatisfactory attitude, and poor medication compliance towards ICS aerosol therapy. The findings may assist healthcare providers in designing educational programs for families with children suffering from wheezing diseases to improve their knowledge and attitudes towards ICS aerosol therapy, promote children’s medication compliance, and eventually improve the prognosis of pediatric wheezing diseases.

Poor knowledge of ICS aerosol therapy is common and has been reported in various studies. Amani et al. found that 70.4% of parents were worried about the adverse effects of ICS, while 29.0% believed that their child would develop a dependency on asthma medications [24]. Another study reported that the majority (79.1%) of patients who did not persist in the ICS treatment were not aware of the anti-inflammatory actions of ICS [13]. Similarly, participants in this study showed insufficient knowledge of ICS therapy, especially a limited understanding of the adverse effects of ICS and medications for wheezing diseases. Therefore, more opportunities for guardians of pediatric patients to learn correct knowledge about wheezing diseases and ICS therapy should be provided.

Participants in this study showed unsatisfactory attitudes and reported poor adherence of their children to ICS therapy, consistent with previous studies [16, 25]. Several factors have been found to be associated with medication non-adherence to the prescribed medicine, including medication cost, fear of medication side effects, and forgetfulness [26, 27]. Patients’ perceptions of disease and ICS and treatment beliefs were also regarded as predictors of adherence to treatment [28]. A recent study in China found that patient beliefs about the necessity of medication had a strong influence on medication adherence of adult patients with asthma [29]. Other risk factors of poor medication included poor adherence in the previous year, younger patients, and patients without exacerbation experiences [30]. It is believed that by improving knowledge about treatments and stimulating positive perceptions towards medicines, better adherence could be achieved [31]. In this study, limited knowledge and negative attitude of guardians towards ICS therapy and having children aged 4–6 years were found to be associated with their children’s poor medication, highlighting the importance of enhancing guardian’s awareness and beliefs towards pediatric wheezing diseases and ICS therapy, especially for preschool patients. Grandparents as guardians were found to be associated with moderate-to-good medication adherence, which might result from their greater childcare experience.

Previous studies found a significant association between poor asthma control and limited parental knowledge of medications, including not knowing their children’s medications (OR = 6.1), and being unaware of the ICS use time (OR = 2.1) [32], while other studies showed no significant association between parental knowledge and disease control [33]. Patients with good medication compliance generally have better control of their asthmatic disease [34]. Although treatment outcomes of the children with wheezing diseases were not collected in the present study, a significant association between knowledge and medication compliance has been found, suggesting that with better knowledge and compliance it might be possible to achieve better outcomes. The relationship between guardians’ knowledge, attitude, medication compliance, and the disease control of pediatric wheezing diseases should be further explored in the future. In particular, future studies could consider incorporating inquiries related to parents’ educational experiences regarding ICS aerosol therapy in order to establish a more comprehensive understanding of the factors influencing parents’ perceptions and knowledge. This would allow for a more nuanced analysis of the links between education, knowledge, and attitudes, providing valuable insights into potential interventions to enhance parental understanding and adherence to recommended treatments. Data related to the duration of ICS use and its impact on compliance might contribute to a more comprehensive understanding of the dynamics between treatment longevity and parental adherence. This information could guide healthcare providers in tailoring education and support initiatives to address potential challenges that may arise with longer-term ICS usage.

There are several limitations in this study. The first limitation is the single-center design; the present results may not be representative of the general child-raising population in China. Moreover, wheezing diseases were less common in children over 6 years old, leading to the additional selection bias. A multicenter, nationwide, large-sample study is still needed. Another limitation is that this study was based on self-reported questionnaire distributed online, with response rate data currently unavailable, limiting reliability of results. In addition, social desirability bias in the participant responses might exist. Questionnaire responses did not check for other sources of knowledge regarding aerosol administration and the rationale for its use received by parents elsewhere that could have a significant impact on their understanding and attitudes towards this form of treatment. Finally, duration of ICS usage was not specified and the treatment outcomes of the wheezing children have not been followed up, and the associations between disease control and patients’ knowledge, attitude, and medication compliance remain to be explored.

## Conclusions

In conclusion, guardians of children with wheezing diseases in China had insufficient knowledge, unsatisfactory attitude, and poor medication compliance towards ICS aerosol therapy. It is recommended that medical staff incorporate information about the adverse effects of ICS, medication, and etiology of wheezing diseases into health education programs and provide a reliable and comprehensive information source for guardians, especially for those with children aged 4–6 years, to enhance their knowledge and attitude towards ICS therapy and improve medication compliance.

## Data Availability

All data generated or analysed during this study are included in this published article.

## Abbreviations

ICS: Inhaled corticosteroids
KMO: Kaiser-Meyer-Olkin
MMAS-8: 8-item Morisky Medication Adherence Scale
ANOVA: Analysis of variance
